# Children’s mental health and the COVID-19 pandemic

**DOI:** 10.1192/j.eurpsy.2021.1746

**Published:** 2021-08-13

**Authors:** D. Molini-Avejonas, P. Pacheco, C. Leal

**Affiliations:** 1 Fonoaudiologia, Universidade de São Paulo, São Paulo, Brazil; 2 Rehabilitation Science, USP, São Paulo, Brazil

**Keywords:** COVID-19, mental health, language, Child development

## Abstract

**Introduction:**

In the midst of a global pandemic with a rising death toll, the children´s mental health can be easily overlooked in the country’s response. But this overlook would have devastating consequences for years to come.

**Objectives:**

The objective of this research is to compare children’s physical and mental development before, during and after the situation of social isolation caused by the pandemic of COVID-19.

**Methods:**

The parents/guardians of 100 children aged between 0 and 5 years and 11 months old were asked to answer questions based on the ASQ-3 (Ages and Stages Questionnaire III), containing questions related to Communication, Gross Motor, Fine Motor, Problem Solving, and Personal-Social and ASQ- SE (Ages and Stages Questionnaires Social-Emotional) addressing issues of self-regulation, compliance, social-communication, adaptive functioning, autonomy, and affect. In addition, behavioral issues related to children’s mental health will be included, such as: aggressiveness, insomnia, lack of appetite, apathy, sadness, tiredness, lack of interest, hyperactivity, manias, tantrum, morning among others. Child development data will be collected before and during quarantine / isolation and later, in a second stage, after the end of social isolation.

**Results:**

The data will be analyzed in order to characterize child behavior before, during and after the period of social isolation, correlating the different areas of child development, especially mental health.

**Conclusions:**

As argued, socially isolated children are at increased risk of health problems in adulthood. Furthermore, studies on social isolation have demonstrated that a lack of social relationships negatively impacts the development of the brain’s structure.

**Disclosure:**

No significant relationships.

